# Assessing exposure to war-related traumatic events in older Vietnamese war survivors

**DOI:** 10.1186/s13031-021-00343-y

**Published:** 2021-03-06

**Authors:** Yvette Young, Kim Korinek, Zachary Zimmer, Tran Khanh Toan

**Affiliations:** 1 Department of Sociology, University of Utah, Salt Lake City, UT USA; 2 Global Aging and Community Initiative, Mount Saint Vincent University, Halifax, Nova Scotia Canada; 3 Family Medicine Department, Hanoi Medical University, Hanoi, Vietnam

**Keywords:** Combat, Nearness to death, Inhospitable conditions, Displacement, Posttraumatic stress disorder, Aging, Vietnam

## Abstract

**Background:**

Though studies measuring war-related stressors and resultant trauma among U.S. military veterans are abundant, few studies address how wartime stressors affect military veterans native to warzones. Even fewer assess the stress exposure and resulting trauma experienced by Vietnamese civilians. This study aimed to construct a scale to quantify wartime stress exposure that is relevant for civilians and military veterans who survived the American War in Vietnam.

**Methods:**

The study analyzed data from a novel source, the Vietnam Health and Aging Study, which surveyed older men and women residing in central and northern Vietnam. We used a combination of exploratory and confirmatory factor analysis with posthoc tests of reliability and validity to derive measures for assessing exposure to war-related traumatic events.

**Results:**

We found that a mix of exposure to death, combat, inhospitable living conditions, and forced displacement comprises the traumatic events that potentially contribute to posttraumatic stress disorder and other mental health problems. However, the particular mix of stressful experiences constituting war trauma differs for civilians, veterans of the formal military, and former members of paramilitary organizations.

**Conclusions:**

These findings suggest the need for distinct but parallel approaches to measuring war-related stressors for populations of veterans and civilians exposed to war in their home countries and the need for greater public attention to the potential lingering trauma of noncombatants.

**Supplementary Information:**

The online version contains supplementary material available at 10.1186/s13031-021-00343-y.

## Background

The last several decades have witnessed mounting empirical evidence demonstrating long-term deleterious health impacts of exposure to war-related traumatic events [1–3]. Mental health outcomes, such as posttraumatic stress disorder (PTSD) and depression, have received particularly intense attention and are perhaps best documented [4–6]. However, it is clear that war trauma associates with an extensive range of other adverse health outcomes and concerns that last throughout life. Lasting effects occur in diverse domains, including health behaviors, functional health and disability, diagnosed chronic conditions, and general global wellbeing. These associations are either a function of the direct impact of the trauma experienced or effects mediated by other psychological causes such as PTSD [7–11].

Assessing precise direct and indirect impacts and possible interventions that may moderate their influences depends greatly on an ability to evaluate wartime experiences. Specifically, it is important to quantify the degree of trauma encountered and categorize traumatic experiences in meaningful ways (e.g., such that they can inform the relationship between war exposure and later-life health). Ideally, such scales assessing exposure could be used by subsequent researchers.

Since the mid-1980s, various approaches and instruments have been advanced to measure wartime exposure and related war stressors [6, 12–14]. Based on the diagnostic criteria for PTSD, particularly Criterion A, which qualifies traumatic exposure, existing scales tend to include items such as those that gauge threats and fear of death, being seriously injured, experiencing sexual violence, witnessing such events or threats to others such as fellow combatants, and experiencing other harmful physical conditions.

The preponderance of studies that have designed measures of war-related traumatic exposure [5, 6, 12–15] focus on American veterans or veterans in other developed western countries fighting wars that occurred far from their communities of origin [16, 17]. Measures adopted to categorize and quantify wartime exposure have less frequently been assessed for use among populations living in places where wars actually ensue. Exposure to traumatic events within the latter groups is particularly salient in *guerilla* warfare situations or contemporary civil conflicts that unfold within villages, neighborhoods, and on roadways within urban and rural developing country settings. In these settings, potentially traumatic exposures might extend to death and injuries occurring among friends, relatives, and neighbors, the experience of being evacuated from one’s home due to fear of oncoming violence, and fears that accrue to civilians and paramilitary personal as well as military combatants.

The case of the American-War fought in Vietnam presents an excellent opportunity to evaluate measures of wartime exposure for the purpose of examining long-term impacts of war. Those who experienced wartime trauma during this period (1965–1975) are moving into older ages, where the incidence and prevalence of chronic conditions and other health problems are heightened. The conflict they experienced was unusually brutal and was experienced by many different groups, including military personnel, paramilitary, and civilian women and men. In Vietnam, exposure among non-military groups has rarely been identified, quantified, and categorized. Doing so requires access to retrospective data collected from cohorts of Vietnamese that lived through the war. This study uses a new and unique data source collected in 2018, the Vietnam Health and Aging Study (VHAS), with just such information. The VHAS surveyed 2447 individuals living in northern and central Vietnam that were differentially exposed to the American War, inquiring about their wartime experiences. Using these items, we aim to construct a scale to evaluate war-related stress exposure among the former members of formal military organizations, paramilitary groups (such as militias and the Youth Shock Brigades), and civilian populations of Vietnam. Such a scale would be valuable for studying the impacts of war-related trauma, specifically long-term health and other life-course outcomes. Service in informal military organizations, such as the Youth Shock Brigades (TNXP) and community militia groups, provided logistical and other support for formal military groups. The TNXP, or youth volunteer force, comprised young volunteers, including young women, who provided various forms of support to the war effort, from provision of weapons and foodstuffs to infrastructure repair and bomb disposal [18]. In many ways, their exposure to dangerous and traumatic events paralleled that of the formal military. However, they were also significantly less likely to exchange fire with the enemy. We compare exposures across these three groups that encountered an assortment of different wartime conditions and experiences.

### Defining traumatic events

Measuring the exposure to traumatic events that precede the experience of trauma and resultant mental and physical health issues requires a clear definition of “traumatic events.” The American Psychiatric Association (APA) defines trauma as “an emotional response to a *terrible event* like an accident, rape or natural disaster” [19]. The “terrible events” of import for this study are war-related traumatic events and stressors.^1^ Researchers have long struggled to understand the precise features of traumatic stressors [20–22]. Weathers and Keane [22] note that crafting a definition of trauma and traumatic events is difficult, in part, due to the many relevant dimensions of stressors, including their “magnitude (which itself varies on several dimensions, e.g., life threat, threat of harm, interpersonal loss … ), complexity, frequency, duration, predictability, and controllability” [22]. As such, most researchers rely on descriptions of traumatic events contained in the diagnostic criteria for PTSD [6, 22, 23]. The clinical definition and diagnostic, found in the current version of the *Diagnostic and Statistical Manual of Mental Disorders* (DSM-5), lays out five criteria for diagnosing PTSD. The first criterion, without which there can be no diagnosis, is exposure to a traumatic event [22, 24, 25]. Specifically, Criterion A requires: “[e] xposure to actual or threatened death, serious injury, or sexual violence,” by directly experiencing or witnessing the traumatic events or learning about the traumatic experiences of family and close friends^2^ [24]. In this paper, we follow previous scholars in relying on the description of traumatic stressors provided in the DSM-5, specifically being wounded, seriously injured, or almost killed; witnessing acts of injury and killing; and learning about the death and injury of family members.^3^ We supplement the events outlined in Criterion A with war-related stressors validated by previous scholars, including engaging in combat duties, exposure to inhospitable conditions, and witnessing the effects of war violence. We also test new stressors for their potential relevance to the Vietnam context.

### Existing warzone stress scales

Two broad research domains have investigated war-related traumatic stressors and how to measure them. One domain focuses on war veterans, while the other centers on refugees.

Prior research into traumatic events experienced by veterans has identified stressors clustering in four dimensions: combat activities, nearness to death and severe injury, moral injury, and inhospitable conditions [12, 13, 26]. However, most researchers did not include all four dimensions in their scales. For many veterans, especially those deployed to combat duties, some exposure to death and serious injury, whether to oneself or others, is practically a foregone conclusion. As such, early scales, like the National Vietnam Veterans Readjustment Study (NVVRS) and Combat Exposure Scale (CES), measured exposure to war-related violence by cataloging combat experiences [6, 14, 27]. However, scholars also recognized that war-related exposure to traumatic events extends beyond mere combat exposure. For example, war-related trauma can extend to morally injurious experiences, or the “harm received to one’s moral center” [28] as a result of “perpetrating, failing to prevent, bearing witness to, or learning about acts that transgress deeply held moral beliefs and expectations” [29]. Personal “moral injury” can occur when one takes another’s life, commits or witnesses the commission of atrocities, or fails to prevent the suffering of civilians or fellow service members [5, 25, 29–31]. Laufer and his colleagues tested a model of war-related trauma that added the moral-injury dimensions of witnessing abusive violence and participating in abusive violence to the typical combat experiences items. This scale was innovative in that it accounted for the guerilla-style of warfare characteristic of the Vietnam War. Laufer’s subjects reported stress related to their inability to “distinguish between noncombatants and the enemy,” “sanctioned acts of brutality,” and the use of “cruel weapons” [15].

Scholars have also documented the role inhospitable living conditions play in trauma exposure [5, 14, 25]. Using their Deployment Risk and Resilience Inventory (DRRI) [13], King et al. [25] found that among American veterans of the Vietnam War, exposure to a “malevolent environment” (i.e., the undesirable food, climate, living conditions, and other chronic, lower-magnitude stressors of warzone deployment) was the most pronounced contributor to PTSD, far surpassing the influence of combat. However, for civilians and local military residents of warzones, the conditions constituting “malevolent” or inhospitable living environs likely differ from those enumerated by veterans of foreign wars, a theme investigated in research on trauma in refugees.

The second domain of trauma research focuses on sources of trauma for refugees, asylees, and displaced persons. This branch of research often omits combat-related inquiries in favor of war-related stressors that anyone might experience, but it adds new dimensions of stressors, including mental injury, extrajudicial operations, and displacement. In addition, this body of work adds breadth to dimensions of trauma investigated with veterans.

A notable omission in measurement scales designed for veterans is the lack of attention to evacuation and other “less than voluntary” migration experiences. This oversight likely stems from the fact that prior research focused primarily on American veterans of foreign wars. However, refugee scholarship informs us that war-related forced migration is concurrent with exposure to other traumatic events and dangers typically associated with warzones. Moreover, forced migration has been associated with subsequent mental distress [32–34] and may connect directly and indirectly to posttraumatic stress. To illustrate the complexity of the relationship between displacement as a traumatic event and mental health, Porter and Haslam [35] describe how refugees experience stress accumulating at multiple different stages of displacement, including preflight, flight, exile, and resettlement or repatriation.

This study integrates the insights of two research domains, evaluating the dimensions of stressful wartime events for use as trauma exposure indicators in northern Vietnamese survivors of the American war. We draw from prior research, combining items from prior scales to investigate these dimensions. We include items related to combat activities, personal and family injury and death, witnessing death and severe injury, moral injury, inhospitable conditions, and displacement. Two recent studies noted that some scales clustering combat activities, injury, and nearness to death into distinct groups demonstrated poor divergent validity [12, 26]. As such, we anticipate that the events may cluster differently than previously thought. We also test whether the dimensionality is consistent across civilians, informal military, and formal military respondents. Finally, the two principal research domains primarily (though not exclusively [17]) study people who no longer reside in the location where they were exposed to conflict, i.e., veterans of foreign wars and refugees resettled outside the warzone. Our study seeks to integrate the two conceptual domains and fill this critical gap in the research [16], investigating the nature of warzone stressors for residents of Vietnam, subgroups of which include civilians, veterans of the formal military, and paramilitaries and others peripherally involved in war efforts.

## Method

### Data and sample

This paper analyzes data from the Vietnam Health and Aging Study (VHAS), collected in face-to-face interviews conducted in 2018. The VHAS was designed to investigate the long-term effects of exposure to war, specifically, the American War, on older adult physical and mental health, overall wellbeing, and mortality. VHAS investigators used a multistage, stratified probability design to sample 2447 men and women aged 60+ in four districts of northern and central Vietnam. This age group was chosen because it encountered the height of the American War (1965–1975) during childhood, adolescence, and early adulthood. The American War in Vietnam was characterized by unprecedented levels of bombing, widespread defoliation, and extensive population displacement [36, 37]. The war also generated considerable military recruitment and mobilization efforts [38]. The four districts sampled —Bavi, Yen Khanh, Dong Hoi, and Bo Trach—were purposively-selected to represent a spectrum of war exposure, as indicated by the intensity of bombings during war years [39, 40].^4^ Within these districts, investigators randomly selected 12 communes, after which they randomly selected 204 individuals from each commune (see 39 for additional details). Men and women with both formal and informal military service were included in the sample, as were nonveterans.

### Measures

Our study’s measures derive from VHAS questions about respondents’ military participation, death and disability among family members, diverse forms of war exposure, and PTSD. The questions are shown in their entirety in Supplementary file 1: Appendix C.

#### Group variable—military participation

Concerning military participation, the survey asked: “Have you ever participated in any military activities?” Response options included: *served in the formal military (Viet Minh, before 1954)*, *served in the formal military (People’s Army of North Vietnam)*, *served in the formal military (Army of the Republic of Vietnam)*, and *served in the Youth Shock Brigade (Thanh niên xung phong or TNXP)*, and *involved in other militia services*. We grouped the formal military service responses into a formal military category and combined service in the TNXP and militia into an informal military category.

#### Death and disability of family members

Reflecting conceptual and operational definitions of traumatic stressors^5^ described above, we included items assessing deaths, severe injury, and disability of family members due to the war [14].^6^ Familial experiences of war-related death and disability were assessed separately for the respondent’s father, mother, brother(s), sister(s), spouse, and children.

#### War exposure variables

The VHAS survey contained question sets assessing four types of trauma exposure referenced in prior studies: nearness to death and severe injury, inhospitable conditions, and combat experiences, including moral injury. VHAS investigators drew from NVVRS [14], DRRI [13], and CES [6] to design war exposure items. Nearness to death items included questions about seeing dead or seriously injured Vietnamese soldiers, foreign soldiers, and civilians; and questions about being injured and knowing people injured or killed in battle. Items gauging exposure to inhospitable conditions included questions documenting displacement due to village bombings or evacuations and questions regarding shortages of clean water and food, inability to sleep due to noise or inhospitable conditions, fearing being injured or killed, and exposure to toxic chemicals, including agent orange.

The combat experience questions—asked only of respondents with formal or informal military experience^7^—inquired about eight combat experiences. These questions assessed how many times the respondent experienced going on patrols, being ambushed, coming under artillery fire, firing at the enemy, being responsible for the death of the enemy, being nearly shot, and having friends shot near them in battle.

#### PTSD variables

The VHAS includes nine questions, drawn from the 20-item PTSD Checklist for the DSM-5 (PCL-5), assessing respondents’ experience of PTSD. These asked whether the respondent had experienced a specified form of stress and how much it bothered them. Questions tapped the respondents’ level of re-experiencing traumatic events, avoiding reminders, emotional numbing, arousal, and anxiety. Only nine questions were incorporated, in part, to reduce respondent burden. In addition, VHAS investigators removed questions that did not translate well, either linguistically or culturally, to the Vietnam context or were politically sensitive. Reduced scales, some with as few as two items, have been previously validated by scholars seeking to apply the scale internationally [41–43]. Lang et al. [41] compared abbreviated scales with the full PCL and clinician diagnoses, finding that longer abbreviated scales performed better than short ones. As such, we used all items available to us in the VHAS.

### Data analysis

The purpose of this study was to establish a scale quantifying stressful war experiences that is valid for veterans of both formal and informal military organizations, as well as civilians in Vietnam. Because the survey combined items from scales previously used solely with veterans assessing both combat and noncombat experiences, with items designed to assess trauma exposure in civilians, it was necessary to evaluate how these questions hang together as indicators of stressful war experiences. To that end, we conducted exploratory factor analysis (EFA), comparing results across civilians, informal military, and formal military, followed by confirmatory factor analysis (CFA). To account for the fact that some items were binary while others contained multiple categories, we recoded all into binary indicators, which reduces method effect biases [44, 45]. To account for the fact that some questions were not asked of civilians, we tested separate models for civilians, informal military, and formal military.

The unique VHAS sampling approach required that we use survey estimation methods and apply sampling weights.^8^ However, many statistical operations limit the application of survey-estimation techniques and sampling weights. In addition, binary variables require special treatment in factor-analytic models, specifically, analysis based on tetrachoric correlations. Thus, our analysis proceeded in three broad steps: 1) preliminary inspection of the tetrachoric correlation matrix, 2) estimation of weighted tetrachoric EFA using the iterated principal factors (IPF)^9^ method with oblique rotation,^10^ 3) examination of survey-adjusted CFA with sampling weights.^11^ All analyses were conducted in Stata version 15.

We undertook step one following DeVellis’ [50] recommendations, who argues that scale items must be highly intercorrelated. Tetrachoric correlations were used to compensate for the artificially-binary structure of our scale items [51]. We engaged in multiple iterations of weighted tetrachoric EFA, step two, to assess item clustering and make a preliminary determination regarding the appropriate number of factors via eigenvalues, scree tests, and parallel analysis. When clear factor structures were not apparent, we compared alternate model specifications, preferring models with the cleanest factor loadings (i.e., strong loadings, no cross-loadings, and multiple items per factor). Table 1 details our retention criteria. Finally, we used survey adjusted, weighted CFA to refine and confirm the relational structure of the factors (e.g., relationship between factors, subdimensions, etc.) and evaluate theoretically-relevant correlations between item error terms.

**Table 1:** Factor Retention and Composition Criteria

	Step	Purpose	Criteria
**Factor (F) Retention**			
Eigenvalue	2	Variance explained by F	≥ 1.0
Scree Test	2	F Strength/relevance	Elbow location [52]
Parallel Test	2	F Strength/relevance	Line intersection
Factor Correlation	2–3	F Distinctiveness	< 0.85 [44, 53]
**Factor Composition**			
Correlation	1	Sufficient association	≥ 0.3 [50]
Loadings	2–3	Item determinacy	≥ 0.4^1^ [50]
Crossloading	2–3	Item distinctiveness	Diff. > 0.5 [46, 50, 51]
# Items per factor	2–3	Model identification	> 2 [44, 50, 51]
Uniqueness	2–3	Proportion unexplained by F	≤ 0.6 [44, 50]

To assess model fit, we evaluated the Kaiser-Meyer-Olkin (KMO) measure of sampling adequacy, root mean squared error of approximation (RMSEA), standardized root mean squared residual (SRMR), and comparative fit index (CFI). Our goodness of fit (GOF) criteria are shown in Table 2. To confirm the appropriateness of scales derived from our factor analysis, we evaluated reliability and validity using coefficient omega,^12^ composite factor reliability (CR),^13^ average variance extracted (AVE),

**Table 2:** Goodness of Fit Criteria

	Step	Purpose	Criteria
**GOF**^**2**^			
KMO	2	Sampling adequacy	≥ 0.7
RMSEA	3	General fit – model parsimony	≤ 0.08 [44, 54]
SRMR	3	Diff. between sample & model	≤ 0.08 [55]
CFI^3^	3	General fit – Comparative fit	≥ 0.95 [54, 55]
**Appropriateness**			
*Reliability*			
Omega	3	Internal reliability	≥ 0.7 [56]
CR	3	Composite reliability	[57]
*Validity*			
AVE	3	Convergent validity	≥ 0.5 or > CR [58, 59]
SC	3	Discriminant validity	AVE > SC [57]
Corr. with PTSD	3	Construct validity^4^	*p* ≤ 0.05 [50, 51, 60]

^1^We retained items with loadings between 0.3 and 0.4 when there were strong theoretical reasons for keeping the item [61]

^2^ Chi-square statistics are not reported as they are not applicable with binary data [44]

^3^ The Tucker-Lewis index (TLI) is not shown as it is prone to bias and model misspecification with binary data [54]

^4^ All of the war-related events included, with the exception of inhospitable conditions, meet Criterion A’s definition of traumatic events, demonstrating content validity. Correlations further show that the events we have assumed to be traumatic are, in fact, associated with trauma, demonstrating construct validity

squared correlations between factors (SC), and the correlation of factors with PTSD. The purpose of each measure and the criteria for establishing reliability or validity are shown in Table 2.

## Results

The analytical sample for this study included 1195 men and 1252 women. In all, 43% of respondents engaged in military activities during the war, with 56% of men and 34% of women reporting military activities.^14^ Nineteen percent were members of the formal military, while 24% participated in informal military activities such as the militia or TNXP. Sixty-nine percent experienced at least one war-specific stressor exposure. Among those with military service, a notable 50% reported no exposure to stressful combat experiences. However, 55% did not serve in the formal military and thus may have served in support rather than combat positions. Table 3 summarizes war-related stressor exposures.

**Table 3:** Descriptive Statistics (percent reporting having had the experience at least once)^a^

	All Rs	Civilians (C)	Informal Mil (I)	Formal Mil (F)	Significant Differences between Groups
**Family Death/Disability**	51.4	51.1	56.6	45.9	I vs. F
Death of family member	32.5	33.7	34.8	26.1	
Disability of family member	27.6	22.1	33.6	27.4	
**Witness Death/Severe Injury**	48.5	35.6	52.4	**80.8**	C vs. F; I vs. F
Saw dead Vietnamese soldiers	35.2	**19.2**	**40.0**	**75.6**	C vs. I; C vs. F; I vs. F
Saw dead foreign soldiers	16.0	**5.8**	**16.3**	**44.9**	C vs. I; C vs. F; I vs. F
Saw dead civilians	40.7	31.6	47.2	**58.8**	C vs. F; I vs. F
Wounded in warzone	11.1	**1.9**	**9.7**	**39.3**	C vs. I; C vs. F; I vs. F
Know injured people	31.8	**20.7**	**39.5**	**54.8**	C vs. I; C vs. F; I vs. F
**Inhospitable Conditions**	59.7	**51.6**	66.0	75.7	C vs. I; C vs. F
Moved due to bombings	22.8	20.5	27.6	23.6	
Moved due to evacuation	21.3	20.1	22.8	23.1	
Shortage of clean water	11.5	7.5	12.3	**22.3**	C vs. F; I vs. F
Food shortage	20.4	17.6	22.0	26.7	
Inability to sleep	36.4	30.3	41.9	47.2	C vs. F
Fear being injured or killed	32.4	30.3	38.9	30.7	
Exposure to toxic chemicals	7.5	**2.2**	**5.2**	**25.5**	C vs. I; C vs. F; I vs. F
**Combat Experiences**^**c**^	21.6	–	**31.0**	**73.5**	I vs. F
Went on military patrols	17.0	–	**21.2**	**63.2**	I vs. F
Was ambushed	11.1	–	**8.6**	**47.7**	I vs. F
Came under artillery fire	12.3	–	**10.9**	**51.1**	I vs. F
Shot at the enemy	10.5	–	**4.9**	**49.2**	I vs. F
Caused death of enemy	6.5	–	**1.8**	**32.0**	I vs. F
Was nearly shot	9.4	–	**10.6**	**36.6**	I vs. F
A friend was shot	11.0	–	**8.8**	**46.9**	I vs. F

^a^Weighted percentages shown

^b^Significance tested via binary logistic regression (survey adjusted, with sampling weights) with marginal predictions and confidence intervals using a significance level of 0.05. Bold indicates that a group is significantly different from all other groups

^c^These questions were only asked of people who participated in military activities. Proportions for informal and formal military are based on veterans only

The weighted tetrachoric correlation matrix showed strong correlations (above 0.3) between all items in the expected groups. Several of the combat and nearness to death items also correlated with shortages of clean water, exposure to toxic chemicals, and experiencing fear of death, indicating potential cross-loading items, requiring clarification via factor analysis. Inhospitable living conditions and war-related migrations also demonstrated the expected correlations plus potential cross-loading correlations. When computed separately for civilians, informal military, and formal military, the correlation matrices showed that correlation patterns varied between the three groups, potentially warranting separate analyses.^15^ We examined these patterns further using weighted tetrachoric EFA with oblique rotation followed by weighted CFA.

In preliminary tests using EFA models, we discovered that although death and disability of family members clustered strongly into two respective factors, the factors were largely unrelated to other stressful war experiences. Additionally, the death and disability of family members items are conceptually and chronologically distinct from the other major war-related stressors (i.e., they may have occurred when the respondent was quite young and could not recall; or may have occurred during earlier wars for which we lack other war stressor measures). As a result, we removed these items from all models.

**Fig. 1:**
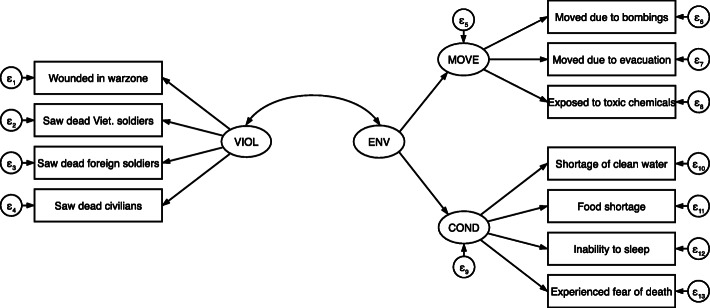
Factor Structure of War-related Traumatic Events for Civilians

Our first set of models, analyzing civilian respondents, yielded three factors: exposure to war violence (VIOL), inhospitable conditions (COND), and war-related migration (MOVE). In EFA, the eigenvalues and the scree test indicated two factors.^16^ However, the two-factor model had cross-loading items indicating either that more factors were needed or that the cross-loading items were not relevant to the factors. We tested models with 1, 2, 3, and 4 factors. The 3-factor model was the only model to meet all factor composition criteria. It also made the most sense theoretically and was confirmed in CFA, with one substantive difference—the COND and MOVE factors were shown to function as sub-dimensions of a broader wartime environment (ENV) factor. Figure 1 shows the relational structure of these factors.

The war violence factor included seeing dead or seriously injured Vietnamese and foreign soldiers, seeing dead or seriously injured civilians, and being wounded in the warzone. *Knowing people who were injured* was removed from the factor during CFA due to its high unique variance. This factor captures experiences related to being the victim of violence, witnessing violence, or witnessing the effects of violence. The inhospitable conditions factor included experiencing shortages of food and clean water, and the inability to sleep due to noise and other inhospitable conditions. The migration factor included three items: *moving due to bombings* and *evacuations*, and *exposure to toxic chemicals*. The wartime environment factor encompasses both displacement and inhospitable living conditions confirming the close relationship between these two sets of items. Table 4 presents factor loadings.

**Table 4:** Factor Loadings for All Models

	Civilians	Informal Mil.	Formal Mil.
**VIOL and COMBAT Factors**	**VIOL**	**COMBAT**	**COMBAT**
Wounded in warzone	0.620	0.617	0.673
Know people who were injured			0.614
Saw dead Vietnamese soldiers	0.991		0.911
Saw dead foreign soldiers	0.823	0.560	0.737
Saw dead civilians	0.816		
Went on combat patrols	—	0.636	0.828
Was attacked/ambushed	—	0.769	0.829
Came under artillery fire	—	0.824	0.889
Shot at the enemy	—		0.898
Caused death of an enemy	—		0.842
Was nearly shot	—	0.828	0.693
Friend was shot near R	—	0.726	0.830
**MOVE and COND Factors**	**MOVE**	**COND**	**COND**
Exposed to toxic chemicals	0.549		
Moved due to bombings	0.792	0.948	
Moved due to evacuation	0.642	0.947	
	**COND**		
Shortage of clean water	0.962	0.615	0.836
Food shortage	0.866	0.552	0.926
Inability to sleep	0.469		0.563
Fear of death/severe injury	0.481		
**ENV Factor**	**ENV**		
COND	0.848	—	—
MOVE	0.797	—	—
N		598	942

The civilian model demonstrated acceptable goodness of fit (KMO = 0.77; RMSEA = 0.05; CFI = .95), and all factors demonstrated acceptable internal reliability (omega ≥ 0.7). All factors also exhibited convergent validity with AVE values greater than 0.5 and discriminant validity. See Table 5 for reliability and validity statistics. Also, correlations between predicted factors and PTSD indicate that the factors are indeed associated with the theoretically-related phenomenon, PTSD, demonstrating construct validity.

**Table 5:** Model Evaluation Statistics — Civilians

	Criteria	VIOL	COND	MOVE	ENV
**Reliability**					
Omega	≥ 0.7	0.89	0.80	0.70	0.87
CR		0.58	0.61	0.60	0.47
**Validity**					
AVE	≥ 0.5 or > CR	0.68	0.53	0.56	0.50
SC	AVE > SC	0.31; 0.35	0.31; 0.26	0.35; 0.26	0.12
Corr. w/ PTSD	p ≤ 0.05	0.31*	0.32*	0.18*	0.31*

**Fig. 2:**
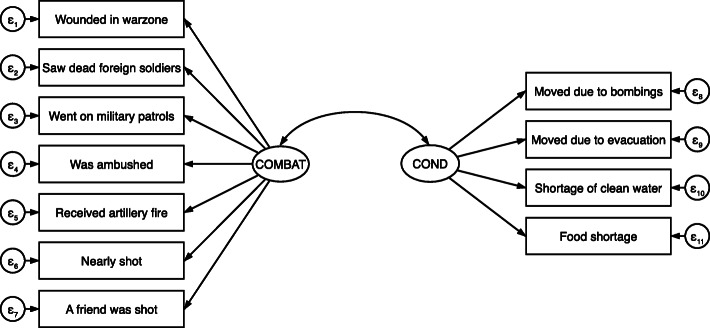
Factor Structure of War-related Traumatic Events for Participants in Informal Military Activities

The second set of models, analyzing informal military respondents, yielded two factors: exposure to combat conditions and related violence (COMBAT) and inhospitable conditions and displacement (COND). In EFA, the eigenvalues and the scree test indicated two factors.^17^ However, the two-factor model had cross-loading items indicating either that more factors were needed or that the cross-loading items were not relevant to the factors. We compared models with 1, 2, 3, and 4 factors. None of the models improved the factor composition statistics. Moreover, the 2-factor model made the most sense theoretically. We revised the 2-factor model, removing *Know people who were injured* due to low loadings and high uniqueness, and removing *Saw dead Vietnamese soldiers* and *Saw dead civilians due* to cross-loading with nearly equivalent values on both factors. At this stage, we retained *Fear of death/severe injury* despite high uniqueness because it had adequate factor loadings, and there are strong theoretical reasons for retaining it. In CFA, we found extremely low factor loadings and high unique variances for *Exposed to toxic chemicals* and *Fear of death/severe injury*. When retaining these items, model fit statistics were poor, and removal of the items improved RMSEA, SRMR, and CFI, raising them to acceptable levels. However, the convergent validity of COMBAT was below acceptable levels (AVE = 0.47). Removal of two additional items (*Shot at the enemy* and *Caused death of an enemy*) exhibiting low equation-level r-squared values improved convergent validity and further improved the model’s goodness of fit. Figure 2 shows the final structure of these factors for respondents with an informal military background.

The combat factor included being wounded in the warzone, going on combat patrols, being ambushed, coming under artillery fire, shooting at the enemy, nearly being shot, and having a friend shot near them in battle. This factor primarily captures experiences related to engaging in the formal activities of war. The inhospitable conditions factor included *moving due to bombing* or *evacuation* and experiencing *food shortages*. Exposure to toxic chemicals, shortages of clean water, and the inability to sleep failed to load on the factor in CFA. See Table 4 for factor loadings.

The informal military model demonstrated acceptable goodness of fit (KMO = 0.78; RMSEA = 0.03; CFI = 0.99), and all factors demonstrated acceptable internal reliability (omega ≥ 0.87). All factors also exhibited convergent validity with AVE values greater than 0.5 and discriminant validity. See Table 6 for reliability and validity statistics. In addition, correlations indicate that the predicted factors were again associated with PTSD, exhibiting construct validity.

**Table 6:** Model Evaluation Statistics — Informal Military Model

	Criteria	COMBAT	COND
**Reliability**			
Omega	≥ 0.7	0.88	0.86
CR		0.50	0.67
**Validity**			
AVE	≥ 0.5 or > CR	0.51	0.62
SC	AVE > SC	0.09	0.09
Corr. w/ PTSD	p ≤ 0.05	0.33*	0.32*

**Fig. 3:**
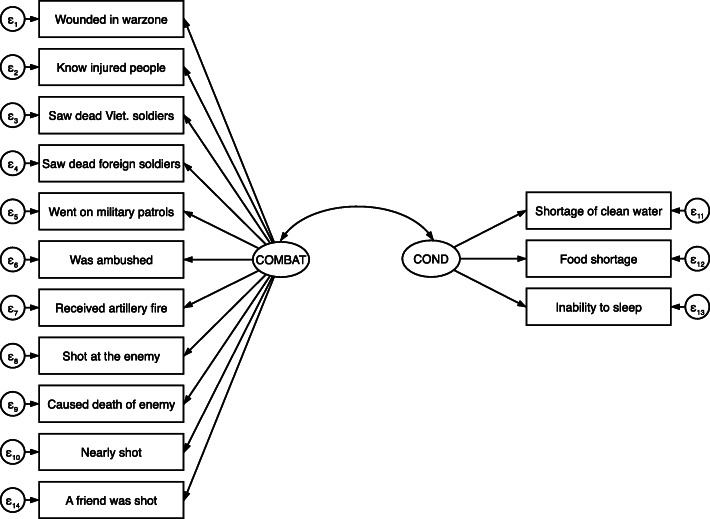
Factor Structure of War-related Traumatic Events for Veterans of the Formal Military

Our final set of models, analyzing formal military respondents, also yielded two factors: exposure to combat conditions and related violence (COMBAT) and inhospitable conditions (COND). In EFA, the eigenvalues and the scree test indicated three factors.^18^ The 3-factor model had one item (*Moved due to bombings*) with a negative uniqueness value, indicating that this solution was a Heywood Case. Factor 3 contained only two items making it “just” identified, thus the possible source of the Heywood solution (Chen 2001). We tested alternate 1- and 2-factor solutions. The 1-factor model exhibited high uniquenesses on all items related to moving and inhospitable conditions, making it a less appropriate solution. In the 2-factor model, *Fear of death/severe injury* failed to load on either factor; it was removed from the final EFA model. While *Exposed to toxic chemicals* had a somewhat low loading on Factor 1 (0.36) and very high uniqueness (0.81), it was retained due to theoretical relevance. In CFA models, *Moved due to bombings* exhibited problematic uniqueness levels,^19^ and when removed, *Moved due to evacuation* had a very high unique variance (0.90). *Saw dead civilians* and *Exposed to toxic chemicals* also demonstrated high unique variances (above 0.80). Removing the four problematic items improved all goodness of fit statistics. Figure 3 shows the relational structure of these factors.

The COMBAT factor included a mix of variables related to being the victim of violence, witnessing violence and its effects, and engaging in combat activities. The inhospitable conditions factor included experiencing shortages of food and clean water, and the inability to sleep due to noise and other inhospitable conditions. However, the factor failed to include war-related displacement. See Table 4 for factor loadings.

The formal military model demonstrated acceptable goodness of fit (KMO = 0.92; RMSEA = 0.04; CFI = 0.98), and all factors demonstrated acceptable internal reliability (omega ≥ 0.83). All factors also exhibited convergent validity with AVE values greater than 0.5 and discriminant validity with factor convergence greater than the squared correlation between factors. Table 7 shows the model’s reliability and validity statistics. Finally, correlations between predicted factors and PTSD indicate that the factors are again associated with PTSD, demonstrating construct validity.

**Table 7:** Model Evaluation Statistics — Formal Military

	Criteria	COMBAT	COND
**Reliability**			
Omega	≥ 0.7	0.94	0.86
CR		0.63	0.60
**Validity**			
AVE	≥ 0.5 or > CR	0.64	0.63
SC	AVE > SC	0.13	0.13
Corr. w/ PTSD	p ≤ 0.05	0.36*	0.29*

## Discussion

The goal of this study was to explore a set of war-related stress exposures to identify items that, together, act as a scale quantifying exposure to war-related traumatic events in a sample of older Vietnamese men and women currently residing in Northern or North Central Vietnam. Our intent was to develop a scale that is valid in the Vietnam context and would allow future researchers to investigate the long-term effects of said war-exposure for civilians, formal military, and paramilitary alike. In pursuing this goal, we integrated insights of two scholarly domains: trauma research focusing on veterans and that focusing on refugees and other civilian residents of warzones. As a result, our study yields novel information on the content of war-related stress for survivors of war in diverse post-conflict contexts outside of the widely studied U.S. veteran population.

Four findings are important to highlight: 1) Some common predictors of PTSD, which may occur during wartime, are not components of war-related trauma exposure; 2) Items from prior instruments did not operate as distinct factors or subscales, rather they clustered with items from other instruments; 3) The contributions of both domains of trauma research—veterans studies and refugee studies—are relevant for studying trauma in residents of warzones; and finally, 4) Civilians, members of militias and other less formalized military organizations, and members of formal military organizations, experience war events differently, indicating the need for distinct measurement approaches.

### War trauma versus general trauma

One common predictor of PTSD, witnessing or learning of the violent or accidental death or severe injury of a family member(s), is a likely occurrence among those who live in warzones. Prior research has shown a strong correlation between these experiences and PTSD or other forms of mental distress [63, 64]. However, in the VHAS sample, the correlation is very weak, ranging from 0.08 to 0.14. Moreover, the DSM-5 enumerates the “violent or accidental” death of family and friends as traumatic events in Criterion A. Our study found that while the death and disability of family members may contribute to PTSD, this form of exposure does not cluster with other war-exposure items. This may indicate that death and disability of family members, however linked to war, is distinct from other war-related trauma exposures and should not be included in scales designed to measure war-related trauma exposure. Alternately, it may reflect the secondary (as opposed to first-hand) nature of these traumas or the fact that they do not co-occur with other exposures in a patterned way. Consequently, researchers should not ignore this predictor of PTSD, especially in contexts where the violence of armed conflict results in loss of life beyond those engaged in the military. However, they should assess its association with PTSD and other mental health indicators separately from other war-related traumatic events.

### Prior instruments and the VHAS population

The VHAS instrument adapted questions from the NVVRS [14], DRRI [13], CES [6], and the PCL-5 while also including questions about injury to oneself, the death and injury of family members, and displacement. This unique composition of items allowed us to identify new factor structures for exposure to traumatic events and conditions in contexts of armed conflict. Prior psychometric testing of war-exposure scales identified three or more factors (depending on the scale), with varying item compositions. For example, U.S. Army and Marine Corps Mental Health Advisory Team’s Combat Experiences Scale (MHAT-CES) identified exposures related to combat environment, direct engagement in combat, and nearness to severe injury or death [26]. However, they acknowledged poor discriminant validity. In other words, the three factors were not discernably distinct from one another. The DRRI identified five war-related factors, including combat experiences, the deployment environment, chemical exposures, deployment concerns, and postbattle experiences. While postbattle experiences largely map onto the MHAT-CES’s nearness to death factor, the other factors diverge considerably. Like the MHAT-CES, the DRRI exhibited poor factor distinctiveness. The VHAS instrument contained items from both of the above scales, supplemented with questions from the NVVRS and questions about wartime displacement. We found that items from previously separate instruments clustered together for this sample, but the precise configurations varied across the civilian, formal military, and paramilitary populations (see Table 8). For example, items from separate factors or subscales, specifically, injury to oneself and exposure to dead or severely injured people, clustered together for civilian respondents. A similar set of items clustered with CES items for both formal and informal military respondents. However, displacement and inhospitable conditions clustered as separate factors for our sample, and the factors exhibited excellent discriminant validity. The restructuring of item clusters from prior scales indicates the need for scholars to draw items from a variety of sources and test factor structures for each new study context.

**Table 8:** Comparison of Factor Structures from Prior Scales

VHAS Items	NVVRS	CES	DDRI	MHAT-CES
Wounded in warzone			Combat Exper.	Direct Engagement
Know people who were injured	X			
Saw dead Vietnamese soldiers			Postbattle Exper.	
Saw dead foreign soldiers			Postbattle Exper.	Near Injury/Death
Saw dead civilians			Postbattle Exper.	
Went on combat patrols		X	Combat Exper.	
Was attacked/ambushed			Combat Exper.	Combat Envir.
Came under artillery fire		X	Combat Exper.	Combat Envir.
Shot at the enemy		X	Combat Exper.	Combat Envir.
Caused death of an enemy			Combat Exper.	Combat Envir.
Was nearly shot		X		Direct Engagement
Friend was shot near R		X	Combat Exper.	Direct Engagement
Exposed to toxic chemicals			Chemical Exposure	
Moved due to bombings				
Moved due to evacuation				
Shortage of clean water	X			
Food shortage			Deployment Env.	
Inability to sleep			Deployment Env.	
Fear of death/severe injury			Deploy. Concerns	

^a^ Items from the NVVRS were used in research, but the psychometric properties of the NVVRS (and its subsections) have never been tested, and its items have not been used in scale form

^b^ CES items are administered as a single, unidimensional scale. It has been shown to have good predictive ability, but the other psychometric properties have not been tested

### Integrating trauma research domains

This study integrated items previously used in two distinct trauma research domains. From studies of trauma in veterans, we incorporated nearness to death and combat exposure. From refugee studies, we included inhospitable conditions and displacement. While prior trauma research with veterans included inhospitable conditions, the studies used conditions perceived to be inhospitable to veterans of foreign wars, such as undesirable food, climate, insects, living conditions, and other chronic, low-magnitude stressors of the deployment environment. These conditions are unlikely to be perceived as inhospitable to populations residing in armed conflict locales. However, psychosocial approaches to the study of war exposure and trauma have noted the importance of “daily stressors,” i.e., stressful social and material conditions, especially as exacerbated by war [65]. Our inclusion of inhospitable conditions such as shortages of food and water, the inability to sleep due to bombings and conflict, and exposure to toxic chemicals supports the findings of scholars taking a psychosocial approach. We found strong support for including these items as potentially traumatic events. Scholars have also noted that post-war “daily stressors” can affect mental health. Future studies should examine the mediating role of post-war stressors for war-exposure and mental health.

We also found support for the inclusion of displacement items, commonly incorporated in refugee studies, for both civilians and members of the informal military. For civilians, displacement clustered with exposure to toxic chemicals, functioning as a subscale within inhospitable environmental conditions, while for the informal military, displacement and inhospitable conditions clustered together in a single factor. This finding likely reflects the co-occurrence of displacement and inhospitable conditions. Prior research has found that inhospitable conditions not only precede or induce displacement they also occur during exile [35]. Conditions of return can also be traumatizing. A study among war survivors in Kosovo investigated why displacement was so frequently listed as a source of trauma [66]. It found that “most people became traumatised after their return to Kosovo because they found their houses and property completely or partially destroyed … They often found that close relatives or friends had been killed or were missing” [66]. Such conditions at each stage of displacement compounded the trauma of displacement itself. These findings indicate the need for researchers to include an array of displacement items, crafting interdisciplinary instruments that draw from both veterans and refugee studies, especially when studying mixed populations (e.g., civilians, formal military, and paramilitary).

### Distinct approaches for separate subpopulations

A central finding of this study is that the items capturing exposure to war-related traumatic events differ for civilians, formal military, and paramilitary. This finding is significant because prior research has not investigated paramilitary groups, such as militias or youth military organizations, as distinct subpopulations uniquely exposed to war events with unique experiences framing their perception of those events. Our analyses found distinct configurations of traumatic events for the three groups.

In interpreting the factor composition for each of these groups, contextual knowledge is critical. For the civilians in the VHAS sample, nearly all of the items available clustered together as indicators of war-related trauma exposure, with one exception: knowing people who were injured. This is best understood in comparison to members of the formal military. Civilians who knew injured people might have been less likely to be traumatized since their acquaintance(s) survived. In contrast, for the formal military, injured acquaintances were likely their fellow soldiers. As such, the members of the military may have experienced something akin to survivor’s guilt or a form of moral injury for failing to prevent the injury of their acquaintance. Additionally, the daily lives of civilians were less frequently impacted by the injury of others. For soldiers, who experienced a daily barrage of diverse traumatic events, knowing others who were injured was likely a more common experience, one that punctuated their other exposures.

Participants in informal military organizations exhibited the most ‘erratic’ configuration of combat/violence items. Though most of the experiences related to military activities and combat duties were elements of the combat factor members of paramilitary organizations, several of the more universally experienced violence-related exposures (i.e., events also experienced by civilians), such as being wounded in the warzone and seeing dead or severely injured foreign soldiers were not. This factor composition may reflect their more limited exposure to combat. As providers of logistical support to the formal military, these respondents were certainly exposed to attacks but were less likely to engage the enemy, thus less likely to be wounded or to see wounded or deceased foreign soldiers [18]. The reduced propensity for exposure to the violent results of engagement with the enemy may increase the salience of these events as indicators of war-related trauma exposure. In contrast, for the formal military, nearly all exposures of the combat-related exposures contribute to the tapestry of war exposure, each adding to the intensity of their exposure.

Finally, while all inhospitable environmental conditions cluster together as traumatic exposures in civilians, albeit in two subfactors, only displacement, and shortages of food and water grouped in a factor for participants in the informal military. King et al. [13] and others have noted the protective effect of military training and preparedness, unit cohesion, and social support for mitigating both exposure and response to war-related traumatic events. As such, the number of inhospitable conditions comprising an inhospitable conditions dimension of war exposure is lower for members of both the informal and formal military. Moreover, for the formal military, the failure of displacement to load on a factor is not unexpected, as they were unlikely to be evacuated but highly likely to be deployed. We also suspect that fearing death failed to load on a factor for both formal and informal military since it may have been a common consequence of their duties, especially combat duties. Thus, combat experiences more accurately reflect their nuanced exposure to the traumatic events of war. Further research should investigate civilian, informal military, and formal military perceptions of their experiences to distinguish how and why the wartime stressors diverge for these groups. Ultimately, the distinct configurations of war exposure factors for these subpopulations speak to the need to recognize and individually analyze subpopulations’ unique exposures to traumatic events. Specifically, in populations residing in warzones, it is crucial to understand the shades of informal military and their roles in the conflict.

One weakness of the current study relates to gender representation across military service subpopulations. In assessing exposure to war-related traumas, it may be important to consider gender as it structures the nature of exposure as well as the consequences of exposure [67]. Although the Vietnamese were heavily mobilized to join the Vietnam War effort, in the VHAS sample, women were still far less likely to be veterans than men. To compensate for gender imbalances in service, the VHAS oversampled women with military service [39]. However, female veterans were less frequently exposed to stressful military experiences than male veterans. This difference in exposure rates likely results from women fulfilling different military roles than men, such as women’s higher prevalence in the Youth Shock Brigades as compared to the North Vietnamese Army. Future studies might further oversample women in various military positions to investigate the intersection of gender and military service and disentangle their distinctive influence upon relevant wartime stressors and the association of wartime stressors with PTSD and other health outcomes.

A second weakness of the study relates to the retrospective nature of the study. The long duration between war exposure and the respondents’ reporting of these events in the VHAS may have impacted recall. Mundane details may fade over time, while some experts believe memories of traumatic events may improve over the years. However, the relationship between trauma and memory is contested and nuanced. Thus, findings based on retrospective accounts should be interpreted with caution. More complete recollections that could add event exposures to a respondent’s account might shift the composition of factors and the resultant scales quantifying war exposure. Despite this limitation, this data presents a unique opportunity for researchers to examine the effects of war exposure, as remembered, on mental health decades later in life.

## Conclusion

This study examined the wartime stress exposure of civilians, veterans of the formal military, and participants in informal military to develop a scale for use among diverse populations in low-income post-conflict countries, such as Vietnam. We validated two factors for each of these populations; however, the composition of these factors varied substantially across the groups.

For analysts seeking to understand the population-wide consequences of war-related trauma exposure, it is essential to incorporate existing war trauma measures drawn from multiple disciplines and develop new measures specific to the social and historical characteristics of the conflict under examination. For example, inhospitable conditions will diverge across different countries, historical eras, and stages of economic development. Additionally, scholars should consider the war tactics used in the conflicts under examination. In a study of the war in Afghanistan, where guerilla warfare tactics have also been used, many previously used questions would be relevant, but new questions might be added to address exposure to improvised explosive devices and other contemporary war tactics [68]. Specific questions related to novel forms of moral injury should also be considered. For example, in the Afghan situation, Taliban fighters regularly use civilians as human shields and disguise themselves as civilians (ibid.). Opposing military forces may repeatedly witness the death of civilians, triggering moral injury.

Finally, while the present study found support for items used in prior instruments, we also found support for new items drawn from other disciplines and items specific to the Vietnam context. We recommend that scholars seeking to measure war-related trauma include items specific to the conflict under investigation, inclusive of exposure to death and severe injury, combat experiences, forced migration, and inhospitable conditions, and with particular attention to subgroups for whom these traumatic exposures might function differently, as they have for the subgroups in our study. This study has contributed to our understanding of diverse wartime experiences, improving our understanding of war-related trauma in the Vietnam context, offering insights for other contexts. Gender, military service, and other social statuses structure exposure to war trauma and reactions to that exposure. Understanding the experiences of distinct subpopulations improves our ability to discern the nature of trauma exposure and craft interventions that address the health and wellbeing of all members of the population.

## Supplementary Information

**Additional file 1.** Appendices.

## Data Availability

The dataset used in the current study are available from the authors upon reasonable request and with completion of data user agreement.

## Abbreviations

APA: American Psychiatric Association
AVE: Average variance extracted
CES: Combat Exposure Scale
CFA: Confirmatory factor analysis
CFI: Comparative fit index
CR: Composite factor reliability
DRRI: Deployment Risk and Resilience Inventory
DSM-5: Diagnostic and Statistical Manual of Mental Disorders – 5th Edition
EFA: Exploratory factor analysis
IPF: Iterated principal factors
GOF: Goodness of fit
KMO: Kaiser-Meyer-Okin
MHAT-CES: Mental Health Advisory Team’s Combat Experiences Scale
NVVRS: National Vietnam Veterans Readjustment Study
PCL-5: PTSD Checklist for DSM-5
PTSD: Postraumatic stress disorder
RMSEA: Root mean squared error of approximation
SC: Squared correlations between factors
SRMR: Standardized root mean squared residual
TNXP: Thanh niên xung phong (Youth Shock Brigades)
VHAS: Vietnam Health and Aging Study

## Footnotes

1 We use the terms *terrible event*, *traumatic event*, and *traumatic stressor* interchangeably throughout the paper*.*

2 See Supplementary file 1: Appendix A for the complete text.

3 We did not include sexual assault due to severe under-reporting. Only 7 respondents, or 0.6% of all women, indicated that they had been sexually assaulted during the war. The question was not asked of men.

4 Bombing intensity data provided by Miguel and Roland [40]. See Figure 1 in Supplementary file 1: Appendix B.

5 See Item 3 of Criterion A in Supplementary file 1: Appendix A.

6 See Supplementary file 1: Appendix C for a question wording and recoding methods for all items.

7 Militia and TNXP members often encountered combat-like conditions. Thus, questions regarding combat experiences were posed to veterans of the formal military, as well as members of the TNXP and other militias.

8 Our survey estimation specified the commune as the primary sampling unit (PSU) and the district as the strata. Survey estimation calculates robust standard errors while accounting for the clustering of errors within strata and PSUs.

9 IPF generates multiple iterations of the squared multiple correlation coefficients to first estimate and then refines the estimation of communalities. Although many scholars recommend maximum likelihood estimation for its handling of missing data, our data contain very few missing values. Moreover, IPF is preferred over maximum likelihood when items are not normally distributed [46, 47]. Since our items are binary we employed IPF rather than maximum likelihood estimation.

10 We used quartimin normalized (oblique) rotation after Stata’s *factor* command. Oblique rotation accounts for the fact that the items and standard errors are correlated. Quartimin “maximizes the variance of the squared loadings within the variables” with oblique rotations [48], based on Harman’s quartimax criterion [49].

11 The models, estimated using Stata’s *sem* command which allows for estimation with binary variables, were run using survey estimation techniques and sampling weights.

12 Coefficient omega estimates the composite reliability using factor loadings and uniqueness, a method which, unlike Cronbach’s alpha, is unbiased when items are congeneric and errors are uncorrelated [56].

13 Composite reliability was calculated using the tetrachoric correlation matrix to ensure its relevance for the binary data.

14 We report survey-adjusted, weighted descriptive statistics in the main text. For comparison, when unweighted, 66.3% of our sample reported some type of military service, with 40.3% reporting formal military service and 26.1% reporting participation in informal military activities.

15 Our preliminary CFA modeled factors by group confirming significant differences between groups.

16 Parallel test values never rose above the eigenvalues, making it uninformative.

17 Parallel test values again failed to rise above the eigenvalues, making the test uninformative.

18 Parallel test values again failed to rise above the eigenvalues, making the test uninformative.

19 The uniqueness was near zero, indicating a Heywood case likely stemming from model misspecification [62]. Removing the item clarified more appropriate solutions.
