# Supplementary material for: Assessing exposure to war-related traumatic events in older Vietnamese war survivors
Source: Confl Health. 2021 Mar 6;15:14. doi: 10.1186/s13031-021-00343-y (PMC7936433; doi:10.1186/s13031-021-00343-y)
Supplement: Supplementary file 1 — Additional file 1. Appendices. [file 13031_2021_343_MOESM1_ESM.docx]

APPENDIX A

*Diagnostic and Statistical Manual of Mental Disorders*, Fifth Ed. Diagnostic criteria for PTSD

| PTSD Diagnostic Criteria—309.81 (F43.10)Posttraumatic Stress Disorder  1. Exposure to actual or threatened death, serious injury, or sexual violence in one (or more) of the following ways:    1. Directly experiencing the traumatic event(s).    2. Witnessing, in person, the event(s) as it occurred to others.    3. Learning that the traumatic event(s) occurred to a close family member or close friend. In cases of actual or threatened death of a family member or friend, the event(s) must have been violent or accidental.    4. Experiencing repeated or extreme exposure to aversive details of the traumatic event(s) (e.g., first responders collecting human remains; police officers repeatedly exposed to details of child abuse). 2. Presence of one (or more) of the following intrusion symptoms associated with the traumatic event(s), beginning after the traumatic event(s) occurred:    1. Recurrent, involuntary, and intrusive distressing memories of the traumatic event(s).    2. Recurrent distressing dreams in which the content and/or affect of the dream are related to the traumatic event(s).    3. Dissociative reactions (e.g., flashbacks) in which the individual feels or acts as if the traumatic event(s) were recurring. (Such reactions may occur on a continuum, with the most extreme expression being a complete loss of awareness of present surroundings.)    4. Intense or prolonged psychological distress at exposure to internal or external cues that symbolize or resemble an aspect of the traumatic event(s).    5. Marked physiological reactions to internal or external cues that symbolize or resemble an aspect of the traumatic event(s). 3. Persistent avoidance of stimuli associated with the traumatic event(s), beginning after the traumatic event(s) occurred, as evidenced by one or both of the following:    1. Avoidance of or efforts to avoid distressing memories, thoughts, or feelings about or closely associated with the traumatic event(s).    2. Avoidance of or efforts to avoid external reminders (people, places, conversations, activities, objects, situations) that arouse distressing memories, thoughts, or feelings about or closely associated with the traumatic event(s). 4. Negative alterations in cognitions and mood associated with the traumatic event(s), beginning or worsening after the traumatic event(s) occurred, as evidenced by two (or more) of the following:    1. Inability to remember an important aspect of the traumatic event(s) (typically due to dissociative amnesia and not to other factors such as head injury, alcohol, or drugs).    2. Persistent and exaggerated negative beliefs or expectations about oneself, others, or the world (e.g., “I am bad,” “No one can be trusted,” “The world is completely dangerous,” “My whole nervous system is permanently ruined”).    3. Persistent, distorted cognitions about the cause or consequences of the traumatic event(s) that lead the individual to blame himself/herself or others.    4. Persistent negative emotional state (e.g., fear, horror, anger, guilt, or shame).    5. Markedly diminished interest or participation in significant activities.    6. Feelings of detachment or estrangement from others.    7. Persistent inability to experience positive emotions (e.g., inability to experience happiness, satisfaction, or loving feelings). 5. Marked alterations in arousal and reactivity associated with the traumatic event(s), beginning or worsening after the traumatic event(s) occurred, as evidenced by two (or more) of the following:    1. Irritable behavior and angry outbursts (with little or no provocation) typically expressed as verbal or physical aggression toward people or objects.    2. Reckless or self-destructive behavior.    3. Hypervigilance.    4. Exaggerated startle response.    5. Problems with concentration.    6. Sleep disturbance (e.g., difficulty falling or staying asleep or restless sleep).  - Duration of the disturbance (Criteria B, C, D, and E) is more than 1 month. - The disturbance causes clinically significant distress or impairment in social, occupational, or other important areas of functioning. - The disturbance is not attributable to the physiological effects of a substance (e.g., medication, alcohol) or another medical condition. |
| --- |

*The complete DSM-5 text (with notes and diagnostic criteria for children) is available online at: https://dsm.psychiatryonline.org/doi/full/10.1176/appi.books.9780890425596.dsm07#BABJAEHE

APPENDIX B


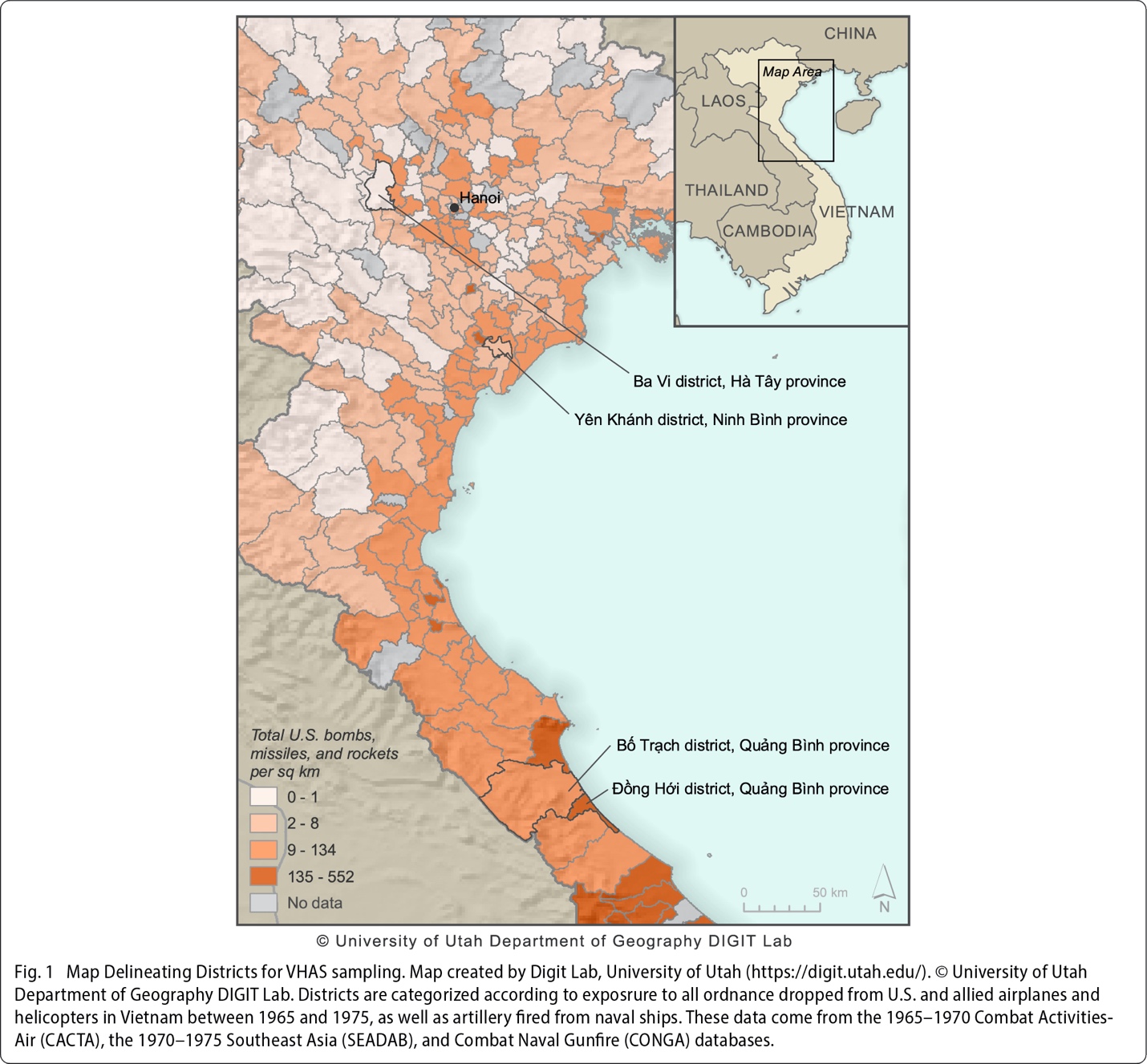


Map previously published in Korinek, et al. 2019. <https://doi.org/10.1186/s12889-019-7680-6>

APPENDIX C

**VHAS Survey Questions, Original Coding, and Recoding**

| **Survey Question** | **Asked of  All Rs?** | **Original Response**  **Categories** | **Recoded Variable Categories** |
| --- | --- | --- | --- |
| **Military Participation** |  |  |  |
| Have you ever participated in any military activities (including TNXP)? | Yes | No/Yes | No coded as *No/Civilian* |
| If yes, have you…   1. Served in the formal military (Viet Minh, before 1954) 2. Served in the formal military (People’s Army of Vietnam) 3. Served in the formal military (Army of the Republic of Vietnam) 4. Served in Youth Shock Brigade (Thanh nien xung phong or TNXP 5. Been involved in the militia |  |  | If yes, **A**, **B**, and **C** coded as *Formal military*. **D** and **E** coded as *Informal military*. |
| **Death/Disability of Family Members** |  |  |  |
| Did any of the following members of your immediate family die in military service, as a civilian casualty, or due to another cause related to war? (*War-related causes include: food shortage, healthcare inadequacy.*)   1. Father 2. Mother 3. Brother(s) 4. Sister(s) 5. Spouse 6. Children | Yes | No  Yes, in military service  Yes, as a civilian casualty  Yes, from another cause related to war (e.g., food shortages or lack of medical care during war),"  Not Applicable^1^  Don’t know/don’t remember^2^ | No/Yes |
| Did any of your family members become disabled due to warzone bombings or shooting?   1. Father 2. Mother 3. Brother(s) 4. Sister(s) 5. Spouse 6. Children | Yes | No/Yes  Not Applicable^1^  Don’t know/don’t remember^2^ | No/Yes |
| **Witness Death and Severe Injury** |  |  |  |
| How often did you see dead or seriously injured foreign soldiers (French, American, Japanese, Cambodian, Chinese…)? | Yes | Never  1 or 2 times/people  3 to 9 times/people  More than 10 times/people | Never/Ever |
| How often did you see dead or seriously injured Vietnamese soldiers (Formal Military)? | Yes | Never  1 or 2 times/people  3 to 9 times/people  More than 10 times/people | Never/Ever |
| How often did you see dead or seriously injured civilians? | Yes | Never  1 or 2 times/people  3 to 9 times/people  More than 10 times/people | Never/Ever |
| Did you know persons who were seriously injured or killed in battle? | Yes | Never  1 or 2 times/people  3 to 9 times/people  More than 10 times/people | Never/Ever |
| Were you ever wounded or injured in the warzone? | Yes | Never  1 or 2 times/people  3 to 9 times/people  More than 10 times/people | Never/Ever |
| **Forced Displacement** |  |  |  |
| How often did your family have to move because home or village was bombed during war (1965-75)? | Yes | Never  1 or 2 times  Often/several times  Very often/many times | Never/Ever |
| How often did your family have to move due to evacuation during war  (1965-75)? | Yes | Never  1 or 2 times  Often/several times  Very often/many times | Never/Ever |
| **Malevolent Conditions** |  |  |  |
| Often during wartime and in the years following, soldiers as well as civilians experience harsh living environments. How often did you encounter the following conditions during the during the following time periods?   - Illness, weakness or discomfort due to shortage of clean water? - Illness, weakness or discomfort due to shortage of food? - Inability to sleep due to noise or inhospitable conditions? - Fear of being injured or killed? - Exposure to toxic chemicals? |  | **a.** Did you experience this?  1. Yes  2. No  8. Refused to answer  9. Don’t know  **b.** If yes, how often during the war (1965-75):  1. Very often/many times  2. Often/several times  3. 1 or 2 times  4. Never  8. Refused to answer  9. Don’t know  **c.** If yes, how often during the years after the war (1976-1985):  1. Very often/many times  2. Often/several times  3. 1 or 2 times  4. Never  8. Refused to answer  9. Don’t know | Never/Ever |
| **Combat Experiences** |  |  |  |
| Considering your military or militia service overall, did you experience these things and how often?   - Did you ever go on combat patrols or perform dangerous duties? - How often did you experience being attacked or ambushed? - How often were you under artillery, rocket, or mortar fire? - How often did you shoot or direct fire at the enemy? - How often were you responsible for the death of an enemy combatant? - How often did you experience a close call, being shot or hit? - How often did you have a buddy who was shot or hit who was near you in battle? | **No** | Never  1 or 2 times  3 to 9 times  More than 10 times | Never/Ever |
| **Posttraumatic Stress** |  |  |  |
| People sometimes have problems in response very stressful experiences during wartime. Given your traumatic experiences during wartime, especially the most stressful experiences/events, did you have the following problems in the last month? If yes, please rate your level of stress.   - Disturbing, and unwanted memories of the stressful experience? - Having strong physical reactions when something reminded you of the stressful experience (for example, heart pounding, trouble breathing, sweating)? - Avoiding memories, thoughts, or feelings related to the stressful experience? - Having strong negative beliefs about yourself, other people, or the world (for example, having thoughts such as: I am bad, there is something seriously wrong with me, no one can be trusted, the world is completely dangerous)? - Loss of interest in activities that you used to enjoy or interest in spending time with other people? - Irritable behavior, angry outbursts, or aggression? - Feeling jumpy or easily startled? - Having difficulty concentrating? - Trouble falling or staying asleep? | Yes | **a.** Did you have the problem  1. Yes  2. No  8. Refused to answer  9. Don’t know  99. Proxy answered  **b.** If yes, did you ever experience this problem?  0. Not at all  1. A little bit  2. Moderately  3. A lot  8. Not applicable  9. Don’t know  99. Proxy answered  **c.** Do you still experience this problem (e.g. in the past year)?  0. Not at all  1. A little bit  2. Moderately  3. A lot  8. Not applicable  9. Don’t know  99. Proxy answered | Items **b** and **c** scored from 0 to 3.  Sum of all points taken to generate raw PTSD score ranging from 0 to 30. |

1 When recoding the measure into a binary variable, we treated "Not applicable" as "No," after verifying that all respondents who replied "Not applicable" had no family at the time of the war.

2 “Don’t know/don’t remember” was coded as “No” for two reasons. First, three of the PTSD symptoms in the VHAS data (and 7 in the PCL-5) require memory of the event occurring. Second, those with multiple “don’t know/don’t remember” responses tended to be older and 85% occurred among respondents who required a proxy to help with some or all of their interview (due to frailty or cognitive difficulties). As such, their responses to 14 of the 19 items were coded as “missing-proxy” and they were omitted from the analysis via listwise deletion.

APPENDIX D

**Supplemental Tables**

Civilians

Table 1. Factor Retention Statistics for Civilian Model

| **Factor Retention Statistics** | **VIOL** | **COND** | **MOVE** |
| --- | --- | --- | --- |
| **EFA** |  |  |  |
| Eigenvalue | 5.25 | 1.57 | 0.86^a^ |
| Scree Test | 2-factors^a^ | | |
| Parallel Test | Uninformative | | |
| Factor Correlations |  |  |  |
| Viol & Cond | 0.36 | 0.36 |  |
| Viol & Move | -0.15 |  | -0.15 |
| Cond & Move |  | 0.21 | 0.21 |

^a^ The eigenvalues and scree test indicated 2 factors. However, the parallel test values never rose above eigenvalues, making it uninformative. The two-factor model had cross-loading items indicating either that more factors were needed or that the cross-loading items were not relevant. We tested models with 1, 2, 3, and 4 factors. The 3-factor model was the only model to meet all factor composition criteria. It also made the most sense theoretically.

Table 2. Weighted Tetrachoric Exploratory Factor Analysis — Civilians

|  | **VIOL** | **COND** | **MOVE** |
| --- | --- | --- | --- |
| Wounded in warzone | **0.608** | 0.009 | -0.013 |
| Know people who were injured | **0.547** | 0.148 | -0.078 |
| Saw dead Vietnamese soldiers | **0.869** | -0.044 | 0.177 |
| Saw dead foreign soldiers | **0.890** | -0.133 | -0.021 |
| Saw dead civilians | **0.707** | 0.093 | 0.250 |
| Moved due to bombings | 0.044 | 0.064 | **0.902** |
| Moved due to evacuation | -0.029 | -0.012 | **0.877** |
| Exposed to toxic chemicals | 0.272 | 0.155 | **0.347** |
| Shortage of clean water | -0.027 | **0.836** | -0.002 |
| Food shortage | -0.114 | **0.957** | -0.004 |
| Inability to sleep | 0.015 | **0.670** | 0.154 |
| Fear of death/severe injury | 0.195 | **0.565** | -0.028 |

Table 3. Confirmatory Factor Analysis — Civilians

|  | **VIOL** | **COND** | **MOVE** | **ENV** |
| --- | --- | --- | --- | --- |
| Wounded in warzone | 0.620 |  |  | — |
| Saw dead Vietnamese soldiers | 0.991 |  |  | — |
| Saw dead foreign soldiers | 0.823 |  |  | — |
| Saw dead civilians | 0.816 |  |  | — |
| Moved due to bombings |  |  | 0.792 |  |
| Moved due to evacuation |  |  | 0.642 |  |
| Exposed to toxic chemicals |  |  | 0.549 |  |
| Shortage of clean water |  | 0.962 |  |  |
| Food shortage |  | 0.866 |  |  |
| Inability to sleep |  | 0.469 |  |  |
| Fear of death/severe injury |  | 0.481 |  |  |
| COND |  |  |  | 0.848 |
| MOVE |  |  |  | 0.797 |
| Covariance (VIOL, ENV) | 0.688 |  |  |  |
| N | 775 |  |  |  |

Table 4. Model Evaluation Statistics — Civilians

|  | **Criteria** | **Statistic** |  |  |  |
| --- | --- | --- | --- | --- | --- |
| **Goodness of Fit** |  |  |  |  |  |
| KMO | ≥ 0.7 | 0.77 |  |  |  |
| RMSEA | ≤ 0.08 | 0.05 |  |  |  |
| SRMR | ≤ 0.08 | 0.04 |  |  |  |
| CFI | ≥ 0.95 | 0.95 |  |  |  |
| CD | — | 0.99 |  |  |  |
| **Appropriateness** |  | **VIOL** | **COND** | **MOVE** | **ENV** |
| *Reliability* |  |  |  |  |  |
| Omega | ≥ 0.7 | 0.89 | 0.80 | 0.70 | 0.87 |
| CR |  | 0.58 | 0.61 | 0.60 | 0.47 |
| *Validity* |  |  |  |  |  |
| AVE | ≥ 0.5 or > CR | 0.68 | 0.53 | 0.56 | 0.50 |
| SC | AVE > SC | 0.31; 0.35 | 0.31; 0.26 | 0.35; 0.26 | 0.12 |
| Corr. w/ PTSD | p ≤ 0.05 | 0.31* | 0.32* | 0.18* | 0.31* |

Informal Military

Table 5. Factor Retention Statistics for Civilian Model

| **Factor Retention Statistics** | **COMBAT** | **COND** |
| --- | --- | --- |
| **EFA** |  |  |
| Eigenvalue | 5.63 | 2.91 |
| Scree Test | 2-factors^a^ | |
| Parallel Test | Uninformative | |
| Factor Correlations | 0.16 | 0.16 |

^a^ The eigenvalues and scree test indicated 2 factors. However, the parallel test values never rose above eigenvalues, making it uninformative. The two-factor model had cross-loading items indicating either that more factors were needed or that the cross-loading items were not relevant. We tested models with 1, 2, 3, and 4 factors. None of the alternate models improved the factor composition statistics. Moreover, the 2-factor model made the most sense theoretically. The 2-factor model was revised, removing *Know people who were injured* due to low loadings and high uniqueness, and removing *Saw dead Vietnamese soldiers* and *Saw dead civilians* due to nearly equivalent cross-loading. At this stage, we retained *Fear of death/severe injury* despite high uniqueness because it had adequate factor loadings, and there are strong theoretical reasons for retaining it.

Table 6. Weighted Tetrachoric Exploratory Factor Analysis

|  | **COMBAT** | **COND** |
| --- | --- | --- |
| Wounded in warzone | **0.488** | 0.297 |
| Saw dead foreign soldiers | **0.530** | 0.194 |
| Went on combat patrols | **0.698** | -0.039 |
| Was attacked/ambushed | **0.751** | 0.023 |
| Came under artillery fire | **0.642** | 0.265 |
| Shot at the enemy | **0.747** | -0.209 |
| Caused death of an enemy | **0.805** | -0.513 |
| Was nearly shot | **0.710** | 0.295 |
| Friend was shot near R | **0.745** | 0.105 |
| Moved due to bombings | 0.264 | **0.664** |
| Moved due to evacuation | 0.250 | **0.673** |
| Exposed to toxic chemicals | 0.025 | **0.610** |
| Shortage of clean water | 0.117 | **0.791** |
| Food shortage | 0.061 | **0.820** |
| Inability to sleep | -0.011 | **0.763** |
| Fear of death/severe injury | -0.186 | **0.399** |

Table 7. Confirmatory Factor Analysis

|  | **COMBAT** | **COND^a^** |
| --- | --- | --- |
| Wounded in warzone | 0.604 |  |
| Went on combat patrols | 0.667 |  |
| Was attacked/ambushed | 0.748 |  |
| Came under artillery fire | 0.798 |  |
| Shot at the enemy | 0.559 |  |
| Was nearly shot | 0.841 |  |
| Friend was shot near R | 0.752 |  |
| Moved due to bombings |  | 0.952 |
| Moved due to evacuation |  | 0.947 |
| Food shortage |  | 0.533 |
| Covariance (COMBAT, COND) | 0.482 |  |
| N | 598 |  |

^a^ CFA results showed an extremely low factor loading and high unique variances for *Exposed to toxic chemicals*, *Fear of death/severe injury*. When retaining these items model fit statistics were poor, and removal of the items improved RMSEA, SRMR, and CFI, raising them to acceptable levels. However, the convergent validity of the COMBAT was below acceptable levels (AVE = 0.47). Removal of two additional items exhibiting low equation-level r-squared values, *Shot at the enemy* and *Caused death of an enemy*, improved convergent validity and further improved the model’s goodness of fit statistics.

Table 8. Model Evaluation Statistics — Informal Military Model

|  | **Criteria** | **Statistic** |  |
| --- | --- | --- | --- |
| **Goodness of Fit** |  |  |  |
| KMO | ≥ 0.7 | 0.78 |  |
| RMSEA | ≤ 0.08 | 0.04 |  |
| SRMR | ≤ 0.08 | 0.04 |  |
| CFI | ≥ 0.95 | 0.97 |  |
| CD | — | 0.91 |  |
| **Appropriateness** |  | **COMBAT** | **COND** |
| *Reliability* |  |  |  |
| Omega | ≥ 0.7 | 0.88 | 0.87 |
| CR |  | 0.51 | 0.53 |
| *Validity* |  |  |  |
| AVE | ≥ 0.5 or > CR | 0.51 | 0.62 |
| SC | AVE > SC | 0.08 | 0.08 |
| Corr. w/ PTSD | p ≤ 0.05 | 0.33* | 0.32* |

Formal Military

Table 9. Factor Retention Statistics for Formal Military

| **Factor Retention Statistics** | **COMBAT** | **COND** |
| --- | --- | --- |
| **EFA (IPF)** |  |  |
| Eigenvalue | 7.73 | 2.03 |
| Scree Test | 3-factors^a^ | |
| Parallel Test | Uninformative | |
| Factor Correlations | 0.74 | 0.74 |

^a^ The eigenvalues and scree test indicated 3 factors. However, the parallel test values never rose above eigenvalues, making it uninformative. The 3-factor model had one item (*Moved due to bombings*) with a negative uniqueness value, indicating that this solution was a Heywood Case. Factor 3 had only 2 items making it underidentified, thus the possible source of the Heywood solution (Chen 2001). We tested 1- and 2-factor models. The 1-factor model exhibited high uniquenesses on all items related to moving and inhospitable conditions. In the 2-factor model *Fear of death/severe injury* failed to load on either factor. In addition, *Exposed to toxic chemicals* had a low loading on factor 1 (0.36), and very high uniqueness (0.81). Both items were removed from the final EFA model, improving model fit.

Table 10. Weighted Tetrachoric Exploratory Factor Analysis

|  | **COMBAT** | **COND** |
| --- | --- | --- |
| Wounded in warzone | **0.659** | 0.038 |
| Know people who were injured | **0.624** | 0.027 |
| Saw dead Vietnamese soldiers | **0.904** | 0.136 |
| Saw dead foreign soldiers | **0.728** | 0.003 |
| Saw dead civilians | **0.448** | 0.286 |
| Went on combat patrols | **0.814** | 0.038 |
| Was attacked/ambushed | **0.828** | 0.007 |
| Came under artillery fire | **0.900** | -0.047 |
| Shot at the enemy | **0.906** | -0.079 |
| Caused death of an enemy | **0.855** | -0.123 |
| Was nearly shot | **0.669** | 0.044 |
| Friend was shot near R | **0.863** | -0.086 |
| Moved due to bombings | -0.176 | **0.841** |
| Moved due to evacuation | -0.179 | **0.818** |
| Shortage of clean water | 0.166 | **0.498** |
| Food shortage | 0.160 | **0.585** |
| Inability to sleep | 0.181 | **0.460** |

Table 11. Confirmatory Factor Analysis

|  | **COMBAT** | **COND** |
| --- | --- | --- |
| Wounded in warzone | 0.669 |  |
| Know people who were injured | 0.616 |  |
| Saw dead Vietnamese soldiers | 0.911 |  |
| Saw dead foreign soldiers | 0.739 |  |
| Went on combat patrols | 0.829 |  |
| Was attacked/ambushed | 0.830 |  |
| Came under artillery fire | 0.889 |  |
| Shot at the enemy | 0.897 |  |
| Caused death of an enemy | 0.841 |  |
| Was nearly shot | 0.689 |  |
| Friend was shot near R | 0.826 |  |
| Shortage of clean water |  | 0.839 |
| Food shortage |  | 0.927 |
| Inability to sleep |  | 0.568 |
| Covariance (COMBAT, COND) | 0.356 |  |
| N | 945 |  |

^a^ CFA results showed low loadings and very high uniquenesses (0.90) for both move variables, removing the variables improved all goodness of fit statistics. However, the convergent validity of both factors was below acceptable levels (AVE = 0.40, 0.44). *Saw dead civilians* and *Exposed to toxic chemicals* also demonstrated high unique variances (above 0.80). Removing the these problematic items improved all goodness of fit statistics and raised convergent validity.

Table 12. Model Evaluation Statistics — Formal Military

|  | **Criteria** | **Statistic** |  |
| --- | --- | --- | --- |
| **Goodness of Fit** |  |  |  |
| KMO | ≥ 0.7 | 0.92 |  |
| RMSEA | ≤ 0.08 | 0.04 |  |
| SRMR | ≤ 0.08 | 0.04 |  |
| CFI | ≥ 0.95 | 0.98 |  |
| CD | — | 0.97 |  |
| **Appropriateness** |  | **COMBAT** | **COND** |
| *Reliability* |  |  |  |
| Omega | ≥ 0.7 | 0.95 | 0.83 |
| CR |  | 0.63 | 0.60 |
| *Validity* |  |  |  |
| AVE | ≥ 0.5 or > CR | 0.64 | 0.63 |
| SC | AVE > SC | 0.04 | 0.04 |
| Corr. w/ PTSD | p ≤ 0.05 | 0.36* | 0.29* |

APPENDIX E

Comparison of Scale Dimensions across the VHAS, NVVRS, CES, DRRI, and MHAT-CES

|  | V-Civ. | V-Inf. | V-Mil. | NVVRS | CES | DDRI | MHAT-CES |
| --- | --- | --- | --- | --- | --- | --- | --- |
| Wounded in warzone | Combat | Combat | Combat |  |  | Combat Exper. | Direct Engagement |
| Know people who were injured |  |  | Combat | X |  |  |  |
| Saw dead Vietnamese soldiers | Combat |  | Combat |  |  | Postbattle Exper. |  |
| Saw dead foreign soldiers | Combat | Combat | Combat |  |  | Postbattle Exper. | Near Injury/Death |
| Saw dead civilians | Combat |  |  |  |  | Postbattle Exper. |  |
| Went on combat patrols | — | Combat | Combat |  | X | Combat Exper. |  |
| Was attacked/ambushed | — | Combat | Combat |  |  | Combat Exper. | Combat Envir. |
| Came under artillery fire | — | Combat | Combat |  | X | Combat Exper. | Combat Envir. |
| Shot at the enemy | — |  | Combat |  | X | Combat Exper. | Combat Envir. |
| Caused death of an enemy | — |  | Combat |  |  | Combat Exper. | Combat Envir. |
| Was nearly shot | — | Combat | Combat |  | X |  | Direct Engagement |
| Friend was shot near R | — | Combat | Combat |  | X | Combat Exper. | Direct Engagement |
| Exposed to toxic chemicals | Move |  |  |  |  | Chemical Exposure |  |
| Moved due to bombings | Move | Conditions |  |  |  |  |  |
| Moved due to evacuation | Move | Conditions |  |  |  |  |  |
| Shortage of clean water | Conditions | Conditions | Conditions | X |  |  |  |
| Food shortage | Conditions | Conditions | Conditions |  |  | Deployment Env. |  |
| Inability to sleep | Conditions |  | Conditions |  |  | Deployment Env. |  |
| Fear of death/severe injury | Conditions |  |  |  |  | Deploy. Concerns |  |

^a^ Items from the NVVRS were used in research, but the psychometric properties of the NVVRS (or any of its subsections) have never been tested and its items have not been used in scale form.

^b^ CES items are administered as a single, unidimensional scale. It has been shown to have good predictive ability, but the other psychometric properties have not been tested.
